# Unmasking the architecture of ant–diaspore networks in the Brazilian Savanna

**DOI:** 10.1371/journal.pone.0201117

**Published:** 2018-08-08

**Authors:** Diego Anjos, Wesley Dáttilo, Kleber Del-Claro

**Affiliations:** 1 Universidade de São Paulo, Programa de Pós-graduação em Entomologia, Ribeirão Preto, SP, Brazil; 2 Red de Ecoetología, Instituto de Ecología AC, Xalapa, Veracruz, Mexico; 3 Universidade Federal de Uberlândia, Uberlândia, MG, Brazil; Universidade de Sao Paulo Faculdade de Filosofia Ciencias e Letras de Ribeirao Preto, BRAZIL

## Abstract

Ant–diaspore interactions are directly related to fruit consumption, seed predation and dispersal, being determinant for the plant fitness. However, although abundant and diversified, these ecological interactions have been neglected in network studies. Understanding the structure of these networks is the first step in preserving these ecological functions. However, describing the network structure is not enough; we need to understand what mechanisms are behind the network patterns. In this study, for the first time, we describe the structure of the ant–diaspore network, considering only the interactions that can benefit plants, separating it into fruit consumption and diaspore removal networks in the Brazilian Savanna. We postulated that ant–diaspore interactions tend to be more specialized in the diaspore removal network compared to the fruit consumption network. Furthermore, we tested whether morphological features, such as size of mandibles of ants and diaspores, could modulate these ecological networks. Overall, we recorded 24 ant and 29 plant species interacting. We found that fruit consumption and diaspore removal networks exhibited similar patterns of interactions (i.e., non-modular), although only the diaspore removal network was nested. The diaspore removal network did not show a more specialized pattern than the fruit consumption network, since both networks consisted of opportunistic interactions. We found that ant mandible and diaspore size does not explain the structure of ecological networks, but in diaspore removal networks the relationship between these morphological traits may explain the pattern of interactions. Thus, we showed that mandible size of ants may have implications on seedling recruitment, suggesting that mandible size can predict possible effects on plant fitness within in diaspore removal networks. Overall, ant–diaspore networks maintain important ecological functions, such as fruit consumption and seed dispersal, which often implies an increase in reproductive success of the plants.

## Introduction

Interactions between ants and diaspores (i.e., seeds or fruits) are very common in the tropics [1–5]. In such environments, most ant–diaspore interactions involve non-myrmecochorous plant species, in which the diaspores do not have elaiosomes [6]. Elaiosomes are lipid-rich appendages on the seed, which that attract ants [7]: they are common on plants in arid and poor-nutrient environments, such as eastern North America, Europe, Australia, South Africa and Northeast Brazil [3,8]. Although non-myrmecochorous plants produce diaspores that lack elaiosomes, they may still have special adaptations for attracting ants, such as their chemical composition and other diaspore traits [9]. Therefore, ants are one of the main seed removers in tropical forests [10], interacting with a diverse range of diaspores, including both myrmecochorous and non-myrmecochorous plant species [4].

Ants and diaspores can interact in different ways, from antagonistic to mutualistic relationships [11]. These insects can have negative effects on diaspores, such as predation, diaspore aggregation and deposition of diaspores in unfavourable germination sites [12]. However, when interacting with diaspores, ants also can increase the reproductive success of the plant in two main ways [13]. Firstly, fruit consumption (as well as seed cleaning) can result in seed scarification, and the ants can release anti-microbial substances [2,14], reducing the mortality of seedlings [15]. Secondly, ants can remove the diaspores from under the parent plant, a process which can be considered diaspore dispersal [16–19], bury them (promoting fire protection) or place them in nutrient-rich sites (e.g., ant nests), increasing the germination rates of the seeds [20–23]. Although these two types of interactions benefit plants, they are very different interactions and can be modulated by different factors such as size and identity of interacting species [9].

At the community level, different ant and plant species interact, creating complex ecological networks where plants and ants are represented as nodes and their interactions are represented as links [24,25]. With the application of network theory in recent years, great advances have been made in the knowledge of the structure of ecological communities [26,27]. However, the use of network theory in the study of ant–diaspore interactions has been neglected (but see [28–31]). As the mechanisms involved in ant–diaspore networks are still not fully understood [30,31], it is necessary to explore both biotic (e.g., the type of interaction, the relative abundance of species and morphological traits) (see [32,33]) and abiotic factors (e.g., seasonality, rainfall, temperature) (see [34–36]). Despite these factors influencing the structure of ecological networks, only a few studies have considered how biotic factors, such as morphological traits, could shape plant–animal interactions (but see [32,37,38]).

Morphological traits of species, in accordance with variations in body size, may be important in structuring ecological networks [27,39–41]. Variation in the mandible size of ants and the sizes of diaspores may be the most important features in structuring ant–diaspore networks, as is the case in plant–pollinator networks, where the sizes of the proboscis and flowers are matched to each other [39,40]. The size of the mandible in ants is directly related to the size of their prey and their capture performance [42]. Additionally, ants should respond to differences in diaspore size, since this can be a major determinants of the outcome of ant–diaspore interactions [9]. Therefore, it is expected that mandible size can be a good predictor of the position of a species in an ant–diaspore network.

Here, we focus on interactions that can benefit plants, then we describe the structure of fruit consumption and diaspore removal networks involving ants in a Brazilian Savanna. To our knowledge, this is the first study to dismantle the ant–diaspore network, considering the fruit consumption and diaspore removal networks separately. We postulated that ant–diaspore interactions tend to be more specialized in the diaspore removal network compared to the fruit consumption network, since ants can feed on virtually any diaspore in the soil but cannot disperse all diaspores due to ecological limitations, such as size and weight. Moreover, we assessed whether the mandible size of ant workers can predict the size of diaspores on which they feed and that they remove, and whether these morphological traits (mandible and diaspore size) explain species roles in the networks. In this case, we expected a positive relationship between mandible size and diaspore size only in the diaspore removal network, like a lock-and-key model, and we expected that these morphological traits can determine the importance of the species in this network.

## Materials and methods

### Study area

Fieldwork was conducted in a Brazilian Savanna reserve, Clube Caça e Pesca Itororó de Uberlândia/CCPIU (18°57'45''S, 48°17'30''W), Uberlândia, Minas Gerais State, Brazil, between June 2015 and May 2016. The Biology Institute of Federal University of Uberlândia has a memorandum of understanding with the CCPIU: an agreement between the board of directors of the CCPIU, and Kleber Del Claro, the director of Research of Federal University of Uberlândia, that enables ecological studies in the area. During fieldwork, we surveyed ant–diaspore interactions in two physiognomy types to increase our sampling effort: cerrado *sensu stricto* and palm swamp areas. The cerrado *sensu stricto* consists of trees 2–8 m tall, with an understory dominated by shrubs, grasses and scattered perennial herbs [43]. Palm swamp environments are characterized by the presence of the palm tree *Mauritia flexuosa* L.f. (Arecales: Arecaceae) and freshwater bodies surrounded by herbaceous vegetation, which mainly consists of species of Cyperaceae, Eriocaulaceae and Poaceae [44]. We chose these two physiognomy types because they have different characteristics and different species diversity, making our sampling more representative in relation to the Brazilian Savanna (see [5]).

### Surveys of ant–diaspore interactions

We established six transects (three in each area), 300 m in length and separated from one another by 150 m. As most ant species forage for distances of less than 150 m, it is unlikely that ant workers that were interacting with diaspores in a transect would be re-sampled on another transect, guaranteeing independence of sampling [45]. We walked each transect monthly (totalling 12 months) and examined the ground 1 m on each side of the transect for ants feeding on or removing fallen diaspores. Transects were surveyed for 1 h 40 min once in the morning (8 to 12 a.m.) and once in the afternoon (1 to 5 p.m.) (totalling 3 h 20min for each transect). The absence of nocturnal observations is a limitation of our study. As only few ant species have nocturnal activity in Brazilian Savanna environments (see [46,47]) and the main seed-removing ant species in the study area has exclusively diurnal activity [48], our observation periods do not make our data incomplete. For instance, Christianini et al. [5] made observations only during the morning and recorded the highest richness of ants and seeds interacting in the Brazilian Savanna. Our observations were always made by the same observer, between days 9 and 12 of each month. Two transects were observed per day, so that six transects were observed on three consecutive days to avoid the influence of major seasonal changes. Thus, we made approximately 230 observation hours (3 h 20 min x 6 transects x 12 months = 230 h 40 min). Each time an ant was observed interacting with a diaspore, the interaction was recorded and the ants and diaspores were collected for identification. Behaviour was classified as a fruit consumption interaction if the ant was in contact with the surface of the diaspore, apparently collecting liquids or removing portions of it (interaction recorded only in cerrado *sensu stricto*), or a removal interaction if the ant was carrying the diaspore (recorded in both physiognomy). Whenever possible, we collected reproductive plant parts to check for correspondence between the interacting diaspore and neighbouring plants with a similar fruit or seed. This material was also used to identify the species, using the Herbarium of Federal University of Uberlândia. All diaspores and plant parts were carefully reviewed by Dr. Cassiano Welker (Federal University of Uberlândia), a specialist in Paniceae s.l. (Poaceae: Panicoideae) and also compared with the collection produced by Belchior et al. [48]. We also recorded the number of ants interacting and identified them according to Baccaro et al. [49].

### Morphological traits of ants and diaspores

Morphometric measurements were taken from the ants using 68 individuals, collected in both areas. The mandible length (see [50]) was obtained using a Nikon SMZ745T stereomicroscope (magnification of 10 x 6 = 60 x), through a UNITRON tablet coupled with the stereomicroscope. We measured the mandible length of each ant worker and lengths of all collected diaspores in the VMS 3.6 program (S1 and S2 Tables, respectively). We used the mean value of mandible length (per ant species) and mean value of diaspore length (per plant species), according to availability: 1–5 ant workers per species; 1–10 diaspores per species. We used diaspore length as a measure that represents the diaspore size, but we know that other measures, such as diaspore weight and shape, are also a good measure for analysing mandible and diaspore size matching. Moreover, these measures may be linked to both diaspore longevity and dispersal distance [51].

### Data analysis

Based on all ant–diaspore interactions collected in our study area, we built interaction matrices ***A***, in which *a*_*ij*_ = 1 if the presence of the ant species *i* on the diaspore species *j* was recorded, and *a*_*ij*_ = 0 otherwise (N = 3 matrices) [52]. Each matrix represented the types of interactions of our study: i) fruit consumption network (i.e., ants feeding on diaspores without diaspore removal); ii) diaspore removal network (i.e., ants removing diaspores to their nests); iii) total network, wherein we considered fruit consumption and diaspore removal networks together. Thus, diaspore removal was a determining factor for the type of interaction (networks) considered here. As the two interactions were never observed at the same time (considering the same ant species), we always considered one or the other type of interaction.

To assess the completeness of the sampling of ant–diaspore interactions, we generated an Interaction Accumulation Curve (IAC) with distinct pairwise interactions as a function of the number of sampled months [53]. For this, we performed non-parametric bootstrapping based on resampling (n = 1000 repetitions) of the presence or absence of a given pairwise interaction in the sampling time [54]. At the species level and for each of the three ant–diaspore networks, we calculated the species strength for both ant and plant species, adapted from Bascompte et al. [55]. Species strength is the sum of dependencies of each species across all its partners (counterpart); species strength quantifies the importance of a given species to species in the other trophic level with which it interacts [55]. For example, the interaction strength of ants was the observed interaction of an ant species on a particular diaspore species (an interaction of ant species *j* to diaspore species *i* divided by the number of interactions of all ant species to diaspore species *i*) and was summed across all diaspore species to determine the relative importance of a particular ant species from the perspective of the interacting plant assemblage. For each network, diaspore and ant species were classified as generalist core or peripheral components according to Dáttilo et al. [56]: G_c_ = (A_i_—A_mean_)/σ_a_ where *A*_*i*_ = mean number of links for a given diaspore or ant species *i*, *A*_*mean*_ = mean number of links for all diaspore or ant species in the network, and *σ*_*a*_ = standard deviation of the number of links for the diaspore or ant species. *G* > 1 are species included in the central core, and *G* < 1 are peripheral species.

We calculated the level of specialization for each of the three ant–diaspore networks using the specialization index *H*_*2*_*’* [57]. This index is derived from the Shannon diversity of networks and is based on the deviation from the expected probability distribution of the interactions. We used binary data to be more conservative and furthermore the ants are social organism being difficult to quantify the frequency [52]. Thus, we used some quantitative metrics such as the degree (assuming all interactions of a species are equiprobable), to know the level at which species interact with more species, being that in this case the frequency does not matter. Therefore, in this study, the specialization is based on the relative degree, instead of calculating the network specialization based on relative frequency. As was adapted to the binary case, P(xi) = 1/d where d is the degree of the focal species. In this case, H(X) = —d*1/d*log(1/d) = -log(1/d) (= I(X)) = log(d), which is the maximal value H(X) can take. Here, H(X) is thus the logarithm of a species degree. We estimated the significance of *H*_*2*_*’* with a Monte Carlo procedure in which 10,000 random matrices were generated using the null model (Patefield algorithm), where marginal totals were identical to those of the observed network [57]. In addition, we tested whether the absolute difference in the observed values of *H*_*2*_*’* between the three empirical ant–diaspore networks was higher than would be expected in simulated networks generated by the null model. To do this, we compared the observed and simulated differences in *H*_*2*_*’* values between: i) fruit consumption and diaspore removal networks (n = 1000 randomizations); ii) fruit consumption and total networks (n = 1000 randomizations); iii) diaspore removal and total networks (n = 1000 randomizations).

We also implemented a second approach that involved the search for non-random patterns in ant–diaspore interactions. Specifically, we evaluated if selective ant species would visit only a subset of diaspore species that were visited by the generalist ant species (i.e., a nested pattern of ant–diaspore interactions). In a nested interaction network, species with a higher number of interactions (i.e., generalist ant species) tend to interact with each other, while species with few interactions (i.e., selective ant species) tend to interact with highly interactive species [58]. We computed the nestedness for each network using the *NODF* metric (Nestedness based on Overlap and Decreasing Fill; Almeida-Neto et al. [59]) in ANINHADO software version 3.0.2 [58]. *NODF* values range from 0 = non-nested to 100 = perfectly nested. Moreover, we tested whether, within each ant–diaspore network, there were groups of ant species that were strongly associated with a particular set of diaspore species, as would be expected in a modular network. For this we used the modularity index (*M*), which estimates the degree to which groups of species (ants and plants) interact more with each other than with species in other groups in the network [60], based on Simulated Annealing (SA) [61] and the software MODULAR version 1.0 [62]. This index ranges from 0 = no subgroups to 1 = totally separated subgroups. We generated random matrices to test the significance of nestedness and modularity according to a null model that probabilistically controls the heterogeneity of interactions, such as the variation in the number of interactions per species (n = 1000 randomizations for each network; [26]).

Finally, to test if there is a relationship between diaspore size and mandible size of the workers, we used a General Linear Model (GLM), using Gaussian distribution with the mandible size as the predictor variable and the diaspore size as the response variable in fruit consumption and diaspore removal networks. Moreover, we tested whether these morphological traits explain species roles in the networks (e.g., species strength), using General Linear Model (GLM) with Gaussian distribution, with the diaspore size as the predictor variable and the strength of plant species as the response variable. In another similar model, we used mandible size as the predictor variable and the strength of ant species as the response variable. Both GLMs were performed with species (plants and ants, respectively) of fruit consumption and diaspore removal networks separately. All GLMs were submitted to residue analysis to verify the adequacy of error distribution [63]. We calculated *H*_*2*_*’* and species strengths (available in the package *bipartite*) [64] and conducted the GLM analysis (available in the base package) using R software version 3.2.3 [65].

## Results

**Fig 1:**
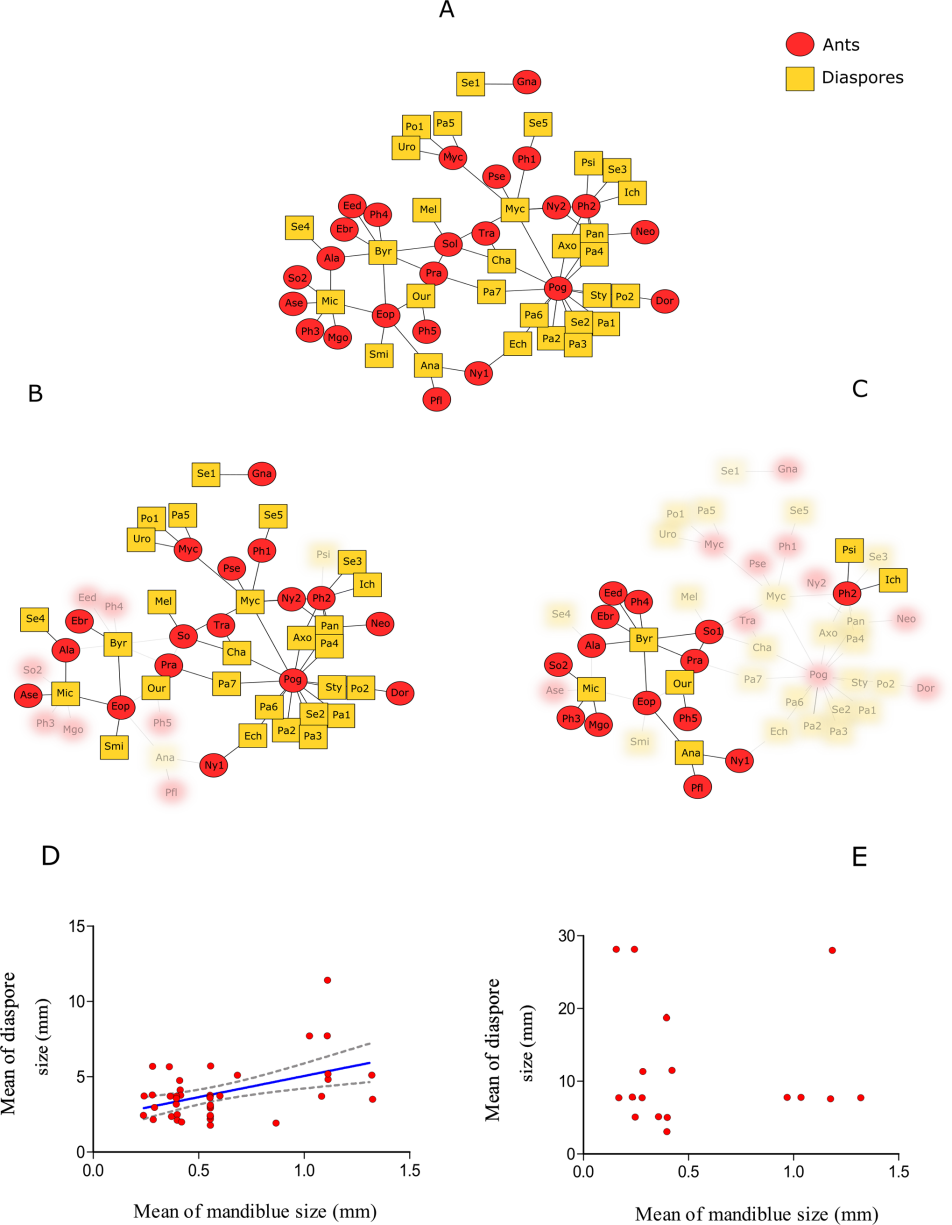
Ant–diaspore networks and the relation between the morphological traits. Ant–diaspore networks and relationship between the mandible size and diaspore size in the Brazilian Savanna involving three types of interactions: A) the total network; B) diaspore removal network; C) fruit consumption network. Within each network, nodes represent one plant species (yellow square) or ant species (red circle) and lines represent ant–diaspore interactions. Relationship between the mandible size of ants and the size of the diaspores on which they D) remove (F_1,43_ = 12.173, p < 0.01) and E) feed (F_1,15_ = 0.210, p = 0.886). The area between dashed lines represents the linear best-fit model (95% confidence intervals). Species name code are in S1 Table and S2 Table.

**Fig 2:**
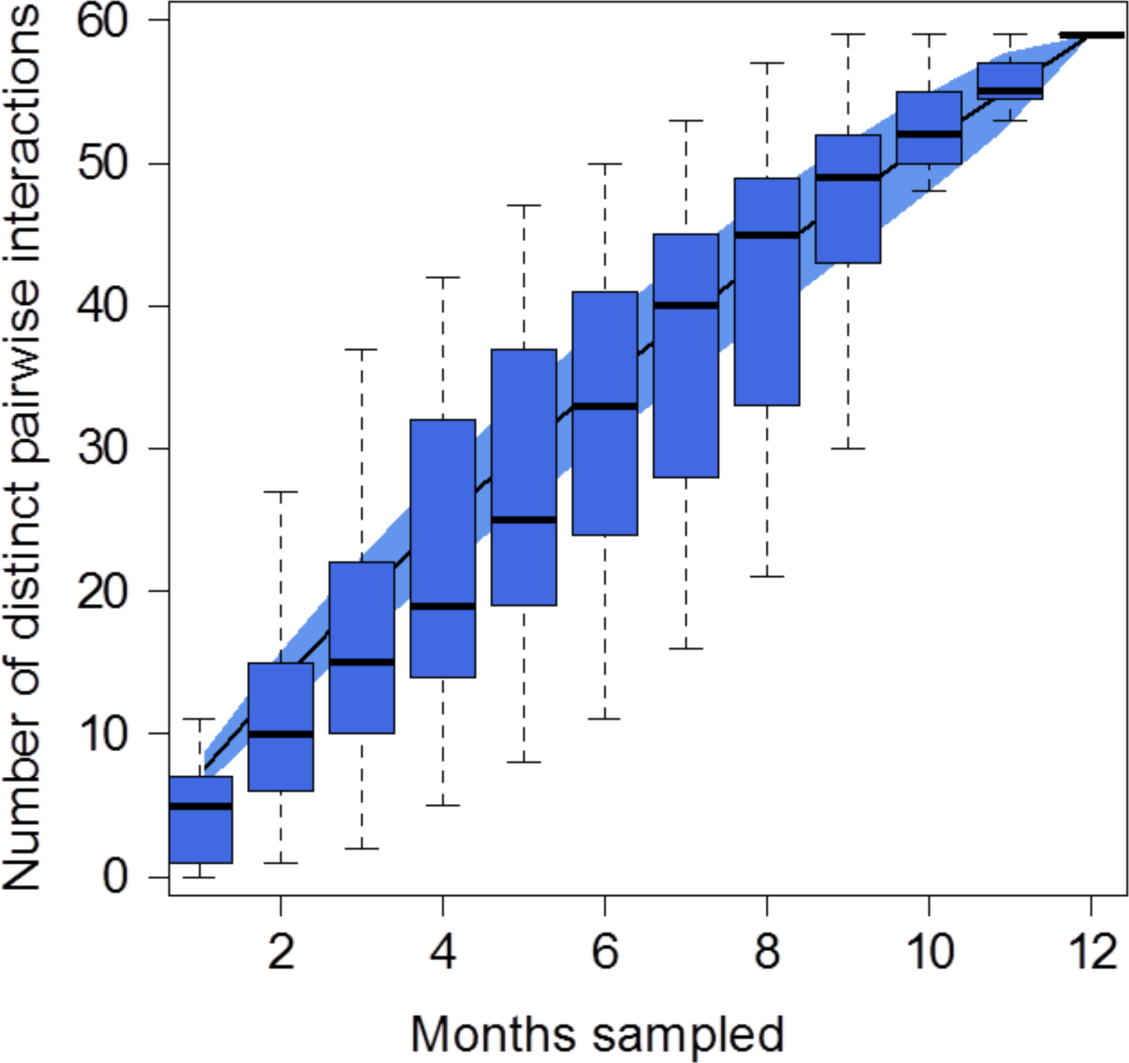
Completeness of the sampling of ant–diaspore interactions. Accumulation curves for distinct pairwise ant–diaspore interactions, recorded over 12 months in the Brazilian Savanna. The error bars represent the 95% confidence intervals.

We recorded 24 ant species, comprised of 14 genera and five subfamilies, with Myrmicinae being the most abundant subfamily. These 24 species of ants removed or fed on 29 plant species, which were distributed among nine families, with Poaceae being the most abundant family (Fig 1A). Only two plant species presented seeds with elaiosomes: Seed sp. 4 and *Microstachys serrulata* (Euphorbiaceae). In the diaspore removal network, we recorded 17 ant species (11 of which were exclusive) carrying diaspores of 27 plant species (23 of which were exclusive; Fig 1B). In the fruit consumption network, we recorded 14 ant species (eight of which were exclusive) feeding on diaspores of six plant species (two of which were exclusive; Fig 1C). *Byrsonima intermedia* (Malpighiaceae), *Miconia albicans* (Melastomataceae), *Ouratea hexasperma* (Ochnaceae) and *Ichnanthus inconstans* (Poaceae) were the plant species observed in both types (fruit consumption and diaspore removal) of ant–diaspore interaction. For the ants, *Atta laevigata* (Myrmicinae), *Ectatomma brunneum* (Ectatomminae), *Ectatomma opaciventre* (Ectatomminae), *Nylanderia* sp. 1 (Myrmicinae), *Pheidole radoszkowskii* (Myrmicinae), *Pheidole* sp. 2 (Myrmicinae) and *Solenopsis* sp. 1 (Myrmicinae) were observed in both types of ant–diaspore interaction. Based on monthly accumulation curves, we recorded 76.6% of the expected pairwise ant–diaspore interactions (observed: 59 interactions; estimated: 77 interactions) (Fig 2).

The plant *B*. *intermedia* (Malpighiaceae) was species that the ants highly depend (illustrated by a higher value of species strength) in the fruit consumption network. Therefore, *B*. *intermedia* was part of the generalist core in the fruit consumption network. In the diaspore removal network, *M*. *albicans* (Melastomataceae), *Panicum cervicatum* (Poaceae), *Microstachys serrulata* (Euphorbiaceae) and *Chamaecrista* sp. 1 (Fabaceae) exhibited higher values of species strength and were part of the generalist core (S3 Table). These plant resources represent ant diet needs and diaspore availability. The ant species *Pheidole* sp. 2 (Myrmicinae) and *Solenopsis* sp. 1 (Myrmicinae) had higher values of species strength in the fruit consumption network and components of the central core of highly interacting species. *Pheidole* sp. 2 (Myrmicinae) and *Pogonomyrmex naegelli* (Myrmicinae) had higher values of species strength in the diaspore removal networks and components of the central core of highly interacting species (S4 Table).

**Fig 3:**
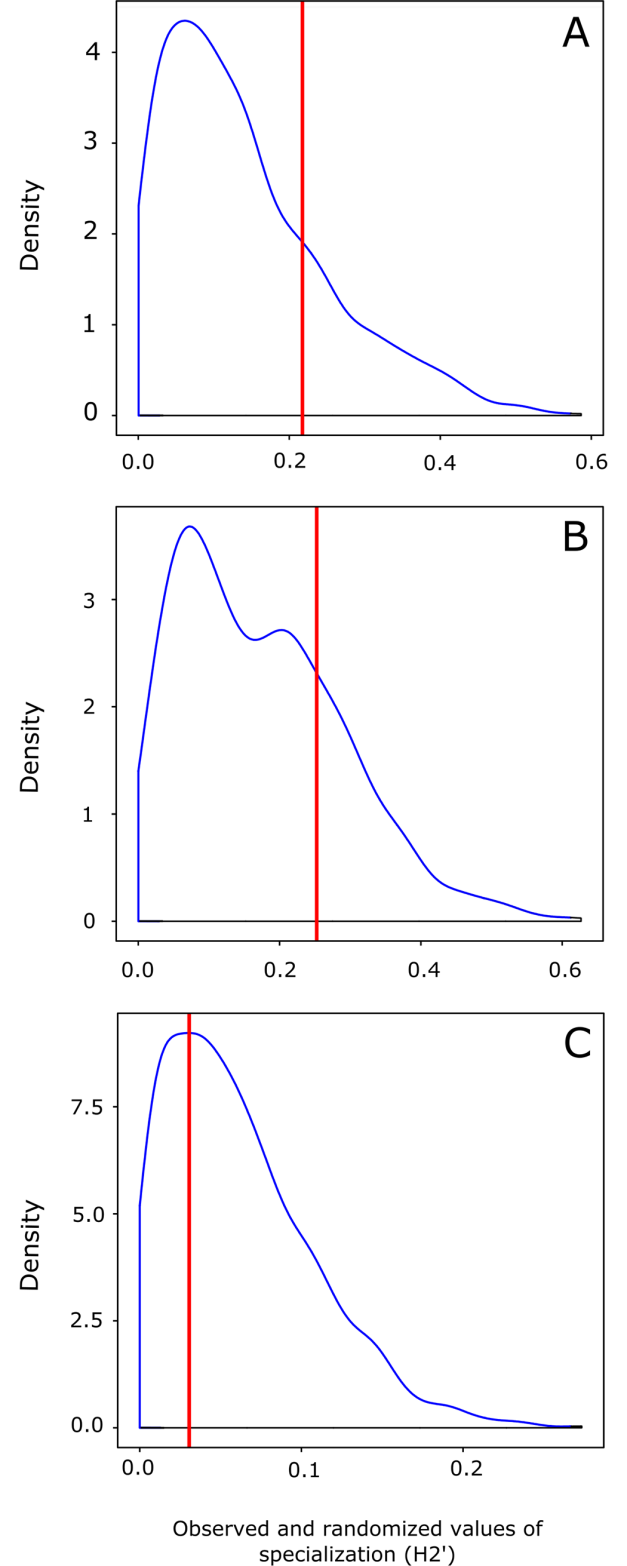
Ant–diaspore specialization. Comparison between the observed (vertical line) differences of *H*_*2*_*’* (specialization) values and the randomized differences of *H*_*2*_*’* values (*Z* test, all *p*-values > 0.05), between A) fruit consumption and diaspore removal networks; B) fruit consumption and total networks; C) diaspore removal and total networks.

We observed that the level of specialization (*H*_*2*_*’*) of the three networks was significantly higher than the null models (total network: *H*_*2*_*’* = 0.31; diaspore removal network: *H*_*2*_*’* = 0.34; fruit consumption network: *H*_*2*_*’* = 0.56; all *p*-values < 0.05). However, the observed differences in *H*_*2*_*’* values did not differ among networks when compared to the randomized differences of *H*_*2*_*’* values (Fig 3; *Z* test, all *p*-values > 0.05), indicating that the three types of network exhibit similar patterns of specialization. In addition, when we evaluated non-random patterns of ant–diaspore interactions within each network, we observed that the diaspore removal network and the total network exhibited significantly nested network topology (diaspore removal network: *NODF* = 19.56, *p* = 0.04; total network: *NODF* = 18.82, *p* = 0.02). This indicates that the interactions recorded for diaspore species that are rarely visited by ants are a cohesive subset of the interactions found on the most visited species. However, we did not find evidence of a nested pattern of species interactions in the fruit consumption network (*NODF* = 13.05, *p* = 0.89). In addition, no network was significantly modular when compared with the null models of ant–diaspore interactions (total network: *M* = 0.61, *p* = 0.34; diaspore removal network: *M* = 0.62, *p* = 0.33; fruit consumption network: *M* = 0.65, *p* = 0.11). This implies that there is no group of ant species that specifically feed on or remove a particular group of plant species.

As expected, we found a relationship between the mandible size of the ants and the size of the diaspore that they remove (F_1,43_ = 12.173, p < 0.01; Fig 1D). We did not observe a relationship between the mandible size of ants and the size of the fruits on which they fed (F_1,15_ = 0.210, p = 0.886; Fig 1E). Furthermore, unlike expected, we did not observe a relationship between morphological traits and species strength in diaspore removal and fruit consumption networks (S5 Table). All analysis and results were taken from the dataset (S6 Table).

## Discussion

Using a network theory approach, we described ant–diaspore networks considering fruit consumption and diaspore removal networks. Considering that most diaspores are grasses (Poaceae) (less attractive to most ants in tropical forests) [9], the number of interactions was considered small (see [1,5]). Ant–diaspore networks exhibited the same patterns of interactions found in most networks involving ants and plants (except ant–myrmecophyte networks) [24] studied at the ecological community level: a nested (except for the fruit consumption network), non-modular pattern of ant–plant interactions and an average level of network specialization [25,36,66–68]. Furthermore, and according to our initial hypothesis, we observed a positive relationship between mandible size and diaspore size only in the diaspore removal network. However, these morphological traits neither modulated the structure of the diaspore removal network nor the fruit consumption network.

Overall, we found that the total, diaspore removal and fruit consumption networks showed similar patterns of specialization, and our hypothesis that diaspore removal networks would be more specialized than fruit consumption networks was not corroborated. Although the three networks demonstrated a higher level of specialization than the null models, indicating that these interactions have a minimum level of specialization, the ants appeared to feed on and remove diaspores in the same way (without any specialization in these interactions). This can be explained by the fact that fruit consumption and removal interactions are likely opportunistic and closely related. At first, the ant–diaspore networks appear to be structured by the availability of diaspores, and the Brazilian Savanna is a seasonal ecosystem where diaspore abundance changes throughout the year [48,69], influencing both types of ant–diaspore interaction (Fig 2). However, when a particular diaspore is available, ants can feed on seeds or remove them, contributing to both ecological networks simultaneously. For example, Myrmicine ants of the genus *Pheidole* are the most common ants that attend diaspores in the tropics, mainly in order to feed (e.g., fruit consumption and seed cleaning) [5,9]. In addition, these ants can also disperse diaspores [9,16,70]. For instance, seedlings of *Miconia* species grew faster and survived better in *Pheidole* spp. refuse piles [71]. Here, *Pheidole* sp. 2 was part of the central core of highly interacting species in fruit consumption and diaspore removal networks. In this way, this ant species may have a double positive role in ant–diaspore networks, as they perform fruit consumption and seed cleaning as well as seed removal and dispersal.

The low richness of myrmecochorous plants (plant species with elaiosomes) found in the study area may also explain why the diaspore removal network was not more specialized than the fruit consumption network. In ant–plant interactions, the myrmecochory is one of the most specialized mutualistic relationships, in which the ants feed on the elaiosome and the plants have their seeds dispersed [4,7,70]. Here, only two myrmecochorous plants, Seed sp. 4 and *Microstachys serrulata* (Euphorbiaceae), were recorded. Nevertheless, *M*. *serrulata* was a plant species more dispersed by ants (higher value of species strength). This is the first description of myrmecochory in the Brazilian Savanna, based on ant–plant interaction studies. However, despite the presence of the elaiosome being uncommon in Brazilian Savanna plants [4], Kuhlmann & Ribeiro [72], recently, recorded several plant species that have these appendages.

Diaspore removal and fruit consumption networks exhibited the same patterns of interactions. However, only diaspore removal and total networks exhibited nested topology. The fruit consumption network probably did not show a nested pattern due to the small number of species and interactions present [24]. Nestedness increases with the complexity (number of interactions) of the network: for a given number of species, communities with more interactions are significantly more nested [26]. We did not find a modular structure in ant–diaspore networks, as in most studies of ant–plant networks (but see [27]). A modular structure would be expected if we had considered all other animal dispersers of these diaspores [73]. Here, for instance, we were probably evaluating a specific module (i.e., removal by ants) within a large diaspore removal network (e.g., meta-networks of dispersal) [73]. Gonzalez-Espinosa & Quintana-Ascencio [74], for instance, considered two species of *Opuntia* (Cactaceae) in Mexico and suggested that ants remove seeds in the same way that dung beetles (see [75]), birds and rodents do [76,77]. The non-modular structure could also be due to the fact that the species of ant and plant had similar traits to each other. For instance, only two ant genera, *Pheidole* and *Ectatomma*, accounted for almost half of the recorded ant species. Likewise, more than half of the identified plant species were from the same plant family, Poaceae, meaning that the species had several traits in common.

Although mandible and diaspore sizes does not determine the importance of species within the fruit consumption and diaspore removal networks, these morphological traits determine the pattern of interactions between species in diaspore removal networks. Therefore, the size of the mandible of ant workers can predict the sizes of diaspores removed by them. The species preferentially interact with the species whose trait combinations match them best, because it allows them to exploit resources most efficiently [78]. Therefore, ant–diaspore interaction may be determined not only by morphological limitation on diaspore removal, but also by the optimization of energy intake when removing them (e.g., fatty acids concentration) [79]. Likewise, phenotypic trait matching is thus a key influence in the effectiveness of plant–animal interactions, where the interaction outcomes depend on close matching [80]. Therefore, in seed dispersal by ants, for instance, with a greater trait-matching fit (e.g., mandible and diaspore size), there is a greater seed removal distance, and the effectiveness of this interaction is greater [9,81]. Moreover, the seed dispersal effectiveness also is influenced by the amount of seed removed, when considering the frequency of interactions, for instance [9,79].

The nestedness pattern could be reinforced by filters or barriers that constrain the occurrence of interactions [37–39,82]. In this way, despite the ability of ants with large mandibles to remove both large and small diaspores [6], we expect that these ants tend to remove larger diaspores more frequently, and ants with small mandibles are limited to removing only small seeds. Nevertheless, this pattern of removal by the ants due to diaspore size has great variation, not generating well-isolated groups (modular pattern). However, Myrmicine ants such as *Pheidole* sp. 2 and *Pogonomyrmex naegelli* (central core of diaspore removal network), which have small mandibles (0.395 ± 0.040 mm; 0.560 ± 0.010 mm, [mean ± SD], respectively), can play an important role in the recruitment and spatial distribution of small-seeded plants (most recorded diaspores are < 6 mm) [6]. Therefore, despite these ant species can prey many seeds that they carry to the nest [71,82], these ants can also disperse diaspores accidentally: (i) seeds that fall along the ant trail, (ii) seeds discarded out of the nest (e.g., in refuse piles outside the nest) and (iii) seeds that grow if the nest is abandoned [83,84]. Although most diaspore species found have been removed by small mandible ants, the large ants often remove diaspores over longer distances than small ants [81]. Thus, diaspores removed by large ants may be more favored, since these ants can benefit seeds by distancing them from the parental plant and escaping the competition under the parental, thus increasing the probability of being in a safe site (distance dispersal hypothesis) [85].

Pizo & Oliveira [9] suggest many ants transport diaspores of a given size limit, and above this limit, however, the ants remove the pulp/aril on the spot without displacing the diaspore. In the fruit consumption network, the mandible size of ants was not a constraining trait since there was no relationship between mandible size and diaspore size. Therefore, ants of similar sizes can feed on diaspores of assorted sizes, and vice versa–, the same fruit can be consumed by ants of different sizes. Small mandible ants such as *Pheidole* spp. can consume small (*Ichnanthus inconstans* (Poaceae): 3.630 ± 0 mm, [mean ± SD]) and large (*Anacardium humile* (Anacardiaceae): 28.090 ± 0 mm, [mean ± SD]) diaspores. In addition, these small ants that usually recruit huge numbers of individuals and dominate large amounts of resources (e.g., large fruits) are able to clean seeds completely [9]. On the other hand, large mandible ants usually remove small pieces of pulp to their nests (Anjos DV *personal observation*). Here, we recorded these two different ant behaviours (*Pheidole flavens* and *Ectatomma opaciventre*, respectively) for the *Anacardium humile* (Anacardiaceae) fruit, the largest diaspore recorded.

In evaluating the two types of ecological networks (fruit consumption and diaspore removal networks), it is suggested that ant species may have a functionality ranking. Here, an interesting result was that ant species considered to be carnivorous, such as *Ectatomma* species [86], seem to be important for ant–diaspore networks, by feeding on and removing diaspores (probably over larger distances than small ants) [79]. In the Brazilian restinga forest, Passos & Oliveira [1] showed that other carnivorous ant species (*Odontomachus chelifer* and *Pachycondyla striata*) were the main diaspore dispersers. In addition, these carnivorous ants can affect the distribution and performance of seedlings of plant species, primarily bird-dispersed [23], or can even promote complementary dispersal [77]. In the Brazilian Savanna, lipid-rich diaspores attract a high-quality guild of dispersers (e.g., large Ponerinae ants), which increase seedling recruitment [77]. However, in open areas of the Brazilian Savanna and in the palm swamp areas where large Ponerinae ants are not common, the *Ectatomma* species can play the functional role of the main diaspore disperser [79]. The *Ectatomma* species can be considered part of a behavioural guild of “high quality dispersers”, since they are large ants that forage individually, carry diaspores to the nest and probably consume only the elaiosome or fruit aril and discard the intact seeds outside the nest [79].

Other ants, such as *Pogonomyrmex naegelli* (Myrmicinae), have unknown roles in ant–diaspore interactions. *Pogonomyrmex naegelli* was in the central core of highly interacting species in the diaspore removal network (exclusive to this network), was the most abundant ant species in this type of ant–diaspore network, and removed diaspores to their nests. This harvester ant is commonly found in open environments of the Brazilian Savanna [48] and some areas with bare soil in the palm swamp habitats. This ant species removes a large amount of diaspores [48] from Poaceae plant species (Fig 1B), the most abundant plant family in the palm swamp areas [44]. However, it is necessary to investigate the true fate of diaspores removed by this ant species and the results of these interactions (Anjos et al., *unpubl*.), because some *Pogonomyrmex* species may have a dual role, acting as predators as well as dispersers of seeds [48,83,84].

In summary, we have described ant–diaspore networks, considering fruit consumption and diaspore removal networks and showed that morphological traits such as mandible and diaspore size do not seem to explain species roles in the networks, but determine the pattern of interactions (e.g., in diaspore removal network). With the inevitable decline of vertebrate dispersers [87], it is expected that ants have an increasingly important role in seed removal [23,77]. Thus, our study produces insights into the ant–diaspore network, since these interactions are still rarely considered by ecologists. Studies that consider ant–diaspore interactions are the first step to assessing the true role of ants in the reproductive success of the plants and how these interactions can also benefit the ant colonies.

## Supporting information

S1 Table Ant species observed in interaction with fallen diaspores in Brazilian Savanna. Data pooled from monthly samples of ant–diaspore interactions recorded along transects at two sites. Ant behavior: FC = fruit consuption through aril or pulp removal on the spot, no displacement; R = remove diaspores > 5 cm. Mandible size ± standard deviation. Standard deviation was done from the number of ants colected, 1 to 5 (according to field records). Key to habitat types: CS = cerrado *sensu stricto*; PS = palm swamp (“vereda”).
(DOCX)

S2 Table Plant diaspores exploited by ants in Brazilian Savanna. Data pooled from monthly samples of ant–diaspore interactions recorded along transects at two sites. Key to habitat types: CS = cerrado *sensu stricto*; PS = palm swamp. Diaspore types were broadly classified as fleshy (with pulp or aril) or dry (no presence of fleshy portion) according to diaspore morphology. Diaspore size (length) (± stardard deviation). Standard deviation was done from the number of seeds colected, 1 to 10 (according to field records). Ant species numbers as in S1 Table.
(DOCX)

S3 Table Ecological importance of plant species. Calculated based on the sum of the level of dependencies from the perspective of the interacting animal assemblage (i.e., species strength). See the text for more information on how estimates of species strength were calculated.
(DOCX)

S4 Table Ecological importance of ant species. Calculated based on the sum of the level of dependencies from the perspective of the interacting plant assemblage (i.e., species strength). See the text for more information on how estimates of species strength were calculated.
(DOCX)

S5 Table Morphological traits explain species roles in the networks. General linear models (GLMs) examining whether the mandible or diaspore size was related to species strength (ant or diaspore/plant species, respectively), considering fruit consumption and diaspore removal network, separately.
(DOCX)

S6 Table Field observations and species traits. Dataset from 2015/2016.
(XLSX)
