# Supplementary material for: Unmasking the architecture of ant–diaspore networks in the Brazilian Savanna
Source: PLoS One. 2018 Aug 8;13(8):e0201117. doi: 10.1371/journal.pone.0201117 (PMC6082530; doi:10.1371/journal.pone.0201117)
Supplement: S1 Table — Data pooled from monthly samples of ant–diaspore interactions recorded along transects at two sites. Ant behavior: FC = fruit consuption through aril or pulp removal on the spot, no displacement; R = remove diaspores > 5 cm. Mandible size ± standard deviation. Standard deviation was done from the number of ants colected, 1 to 5 (according to field records). Key to habitat types: CS = cerrado sensu stricto; PS = palm swamp (“vereda”). (DOCX) [file pone.0201117.s001.docx]

**S1 Table**.

| **Ant subfamily and species (name code)** | **Ant behavior** | | **Mandible size** | **Habitat** |
| --- | --- | --- | --- | --- |
| Ectatomminae | |  |  |  |
| 1. *Ectatomma brunneum* Smith (Ebr) | | FC, R | 1.033 ± 0.070 | CS |
| 1. *Ectatomma edentatum* Roger (Eed) | | FC | 0.975 ± 0.205 | CS |
| 1. *Ectatomma opaciventre* Roger (Eop) | | FC, R | 1.118 ± 0.096 | CS |
| 1. *Gnamptogenys striatula* Mayr (Gna)   Dolichoderinae | | R | 0.860* | PS |
| 1. *Dorymyrmex* pr *pyramicus* (Dor) | | R | 0.290* | PS |
| Ponerinae | |  |  |  |
| 1. *Neoponera apicalis* Latreille (Neo) | | R | 1.080* | PS |
| Myrmicinae | |  |  |  |
| 1. *Atta laevigata* (Smith) (Ala) | | FC, R | 1.328 ± 0.268 | CS |
| 1. *Atta sexdens* Forel (Ase) | | R | 0.680* | CS |
| 1. *Mycetagroicus cerradensis* Brandão & Mayhé-Nunes (Myc) | | R | 0.410* | CS |
| 1. *Mycocepurus goeldii* Santschi (Mgo) | | R | 0.395 ± 0.021 | CS |
| 1. Nylanderia sp. 1 (Ny1) | | FC, R | 0.240± 0.018 | CS, PS |
| 1. *Nylanderia* sp. 2 (Ny2) | | R | 0.360 ± 0.035 | PS |
| 1. *Pheidole flavens* Roger (Pfl) | | FC | 0.150* | CS |
| 1. *Pheidole radoszkowskii* Mayr (Pra) | | FC, R | 0.240 ± 0.020 | CS |
| 1. *Pheidole* sp. 1 (Ph1) | | R | 0.370 ± 0.011 | CS, PS |
| 1. *Pheidole* sp. 2 (Ph2) | | FC, R | 0.395 ± 0.040 | CS |
| 1. *Pheidole* sp. 3 (Ph3) | | FC | 0.357* | CS |
| 1. *Pheidole* sp. 4 (Ph4) | | FC | 0.175* | CS |
| 1. *Pheidole* sp. 5 (Ph5) | | FC | 0.423 ± 0.028 | CS |
| 1. *Pogonomyrmex naegelli* Emry (Pog) | | R | 0.560 ± 0.010 | CS, PS |
| 1. *Solenopsis* sp. 1 (So1) | | FC, R | 0.280 ± 0.030 | CS, PS |
| 1. *Solenopsis* sp. 2 (So2) | | FC | 0.250* | CS |
| 1. *Trachymyrmex* gr *urichi* sp. 1 (Tra) | | R | 0.360* | CS |
| Pseudomyrmicinae | |  |  |  |
| 1. *Pseudomyrmex termitarius* Smith (Pse) | | R | 0.600 ± 0.021 | CS, PS |

*Individuals that were only recorded once.
