# Supplementary material for: Unmasking the architecture of ant–diaspore networks in the Brazilian Savanna
Source: PLoS One. 2018 Aug 8;13(8):e0201117. doi: 10.1371/journal.pone.0201117 (PMC6082530; doi:10.1371/journal.pone.0201117)
Supplement: S2 Table — Data pooled from monthly samples of ant–diaspore interactions recorded along transects at two sites. Key to habitat types: CS = cerrado sensu stricto; PS = palm swamp. Diaspore types were broadly classified as fleshy (with pulp or aril) or dry (no presence of fleshy portion) according to diaspore morphology. Diaspore size (length) (± stardard deviation). Standard deviation was done from the number of seeds colected, 1 to 10 (according to field records). Ant species numbers as in S1 Table. (DOCX) [file pone.0201117.s002.docx]

**S2 Table.**

| **Plant family and species (name code)** | **Habitat** | **Diaspore type** | | **Diaspore size**  **(mm)** | **Ant species** |
| --- | --- | --- | --- | --- | --- |
| Anacardiaceae  *Anacardium humile* St. Hil (Ana) | CS | Fleshy | | 28.090* | 3, 11, 15 |
| Euphorbiaceae  *Microstachys serrulata* (Mart.) Müll. Arg. (Myc) | CS | Dry | | 3.750 ± 0.177 | 9, 11, 15, 16, 20, 21, 24 |
| Fabaceae  *Chamaecrista* sp. 1 (Cha) | CS | Dry | | 5.700* | 20, 21, 23 |
| *Stylosanthes gracilis* Kunth (Sty)  Inderterminate | PS | Dry | | 2.280 ± 0.106 | 20 |
| Seed sp. 1 (Se1) | PS | Dry | | 1.850* | 4 |
| Seed sp. 2 (Se2) | PS | Dry | | 2.900* | 20 |
| Seed sp. 3 (Se3) | CS | Dry | | 5.400* | 16 |
| Seed sp. 4 (Se4) | CS | Dry | | 6.860* | 7 |
| Seed sp. 5 (Se5)  Malpighiaceae | CS | Dry | | 2.370* | 15 |
| *Byrsonima intermedia* A. Juss. (Byr) | CS | Fleshy | | 7.704 ± 0.312 | 1, 3 |
| Melastomataceae |  | |  |  |  |
| *Miconia albicans* (Sw.) Triana (Mic) | CS | Fleshy | | 6.000 ± 0.453 | 3, 7, 8 |
| Myrtaceae  *Psidium* sp. 1 (Psi) | CS | Fleshy | | 18.950* | 16 |
| Ochnaceae  *Ouratea hexasperma* (A.St.-Hil.) Baill. (Our) | CS | Fleshy | | 11.410 ± 2.330 | 3, 19, 21 |
| Poaceae  *Axonopus pressus* (Nees ex Steud.) (Axo) | CS | Dry | | 2.150 * | 16, 20 |
| *Echinolaena inflexa* (Poir.) (Ech) | CS, PS | Dry | | 3.707 ± 0.282 | 11, 20 |
| *Ichnanthus inconstans* (Trin. ex Nees) (Ich) | CS | Dry | | 3.138 ± 0.346 | 16 |
| *Melinis minutiflora* P. Beauv. (Mel) | CS | Dry | | 2.140* | 16 |
| *Paniceae* s.l. sp. 1 (Pa1) | PS | Dry | | 1.746 ± 0.039 | 20 |
| *Paniceae* s.l. sp. 2 (Pa2) | PS | Dry | | 3.510* | 20 |
| *Paniceae* s.l. sp. 3 (Pa3) | PS | Dry | | 3.490* | 20 |
| *Paniceae* s.l. sp. 4 (Pa4) | CS, PS | Dry | | 2.240 ± 0.307 | 16, 20 |
| *Paniceae* s.l. sp. 5 (Pa5) | CS | Dry | | 4.170* | 9 |
| *Paniceae* s.l. sp. 6 (Pa6) | PS | Dry | | 3.110* | 20 |
| *Paniceae* s.l. sp. 7 (Pa7) | CS | Dry | | 2.430* | 14 |
| *Panicum cervicatum* Chase (Pan) | CS, PS | Dry | | 3.690 ± 0.218 | 6, 12, 16, 20 |
| *Poaceae* sp. 1 (Po1) | CS | Dry | | 2.060* | 9 |
| *Poaceae* sp. 2 (Po2) | PS | Dry | | 2.930* | 5, 20 |
| *Urochloa* sp. 1 (Uro) | CS | Dry | | 4.745 ± 0.007 | 9 |
| Smilacaceae |  |  | |  |  |
| *Smilax brasiliensis* Spreng. (Smi) | CS | Fleshy | | 4.810* | 3 |

*Individuals that were only recorded once.
