# Supplementary material for: Unmasking the architecture of ant–diaspore networks in the Brazilian Savanna
Source: PLoS One. 2018 Aug 8;13(8):e0201117. doi: 10.1371/journal.pone.0201117 (PMC6082530; doi:10.1371/journal.pone.0201117)
Supplement: S3 Table — Calculated based on the sum of the level of dependencies from the perspective of the interacting animal assemblage (i.e., species strength). See the text for more information on how estimates of species strength were calculated. (DOCX) [file pone.0201117.s003.docx]

**S3 Table.**

| **Plant species (Family)** | **Fruit consumption network** | **Diaspore removal network** | **Total network** |
| --- | --- | --- | --- |
| *Anacardium humile* (Anacardiaceae) | 2.51 | - | 1.64 |
| *Axonopus pressus* (Poaceae) | - | 0.17 | 0.14 |
| *Byrsonima intermedia* (Malpighiaceae) | 6.12* | 1.41 | 4.72* |
| *Chamaecrista* sp1 (Fabaceae) | - | 1.43* | 1.22 |
| *Echinolaena inflexa* (Poaceae) | - | 1.11 | 0.61 |
| *Ichnanthus inconstans* (Poaceae) | 0.50 | 0.28 | 0.33 |
| *Melinis minutiflora* (Poaceae) | - | 0.33 | 0.12 |
| *Miconia albicans* (Melastomataceae) | 3.13 | 1.86* | 4.64* |
| *Microstachys serrulata* (Euphorbiaceae) | - | 2.87* | 2.63* |
| *Ouratea hexasperma* (Ochnaceae) | 1.42 | 0.21 | 1.39 |
| Paniceae sl sp1 (Poaceae) | - | 0.16 | 0.16 |
| Paniceae sl sp2 (Poaceae) | - | 0.03 | 0.03 |
| Paniceae sl sp3 (Poaceae) | - | 0.03 | 0.03 |
| Paniceae sl sp4 (Poaceae) | - | 0.21 | 0.17 |
| Paniceae sl sp5 (Poaceae) | - | 0.21 | 0.21 |
| Paniceae sl sp6 (Poaceae) | - | 0.03 | 0.03 |
| Paniceae sl sp7 (Poaceae) | - | 1.01 | 0.33 |
| *Panicum cervicatum* (Poaceae) | - | 1.77* | 1.74* |
| Poaceae sp1 (Poaceae) | - | 0.21 | 0.21 |
| Poaceae sp2 (Poaceae) | - | 1.03 | 1.03 |
| *Psidium* sp1 (Myrtaceae) | 0.51 | - | 0.11 |
| Seed sp1 | - | 1.01 | 1.01 |
| Seed sp2 | - | 0.51 | 0.51 |
| Seed sp3 | - | 0.03 | 0.03 |
| Seed sp4 | - | 0.14 | 0.11 |
| Seed sp5 | - | 0.33 | 0.25 |
| *Smilax brasiliensis* (Smilacaceae) | - | 0.21 | 0.14 |
| *Stylosanthes gracilis* (Fabaceae) | - | 0.03 | 0.03 |
| *Urochloa* sp1 (Poaceae) | - | 0.41 | 0.41 |

Asterisks (*) denote plant species that were present in the central core of highly interacting species.
