# Supplementary material for: Unmasking the architecture of ant–diaspore networks in the Brazilian Savanna
Source: PLoS One. 2018 Aug 8;13(8):e0201117. doi: 10.1371/journal.pone.0201117 (PMC6082530; doi:10.1371/journal.pone.0201117)
Supplement: S4 Table — Calculated based on the sum of the level of dependencies from the perspective of the interacting plant assemblage (i.e., species strength). See the text for more information on how estimates of species strength were calculated. (DOCX) [file pone.0201117.s004.docx]

**S4 Table.**

| **Ant species (Subfamily)** | **Fruit consumption network** | **Diaspore removal network** | **Total network** |
| --- | --- | --- | --- |
| *Atta laevigata* (Myrmicinae) | 0.08 | 1.51 | 1.35 |
| *Atta sexdens* (Myrmicinae) | - | 0.25 | 0.14 |
| *Dorymyrmex* pr *pyramicus* (Dolichoderinae) | - | 0.51 | 0.51 |
| *Ectatomma brunneum* (Ectatomminae) | 0.25 | 0.33 | 0.26 |
| *Ectatomma edentatum* (Ectatomminae) | 0.08 | - | 0.06 |
| *Ectatomma opaciventre* (Ectatomminae) | 0.41 | 2.91 | 1.92 |
| *Gnamptogenys striatula* (Ectatomminae) | - | 1.01 | 1.01 |
| *Mycetagroicus cerradensis* (Myrmicinae) | - | 3.01 | 3.07 |
| *Mycocepurus goeldii* (Myrmicinae) | 0.33 | - | 0.14 |
| *Neoponera apicalis* (Ponerinae) | - | 0.14 | 0.14 |
| *Nylanderia* sp1 (Myrmicinae) | 0.33 | 0.25 | 0.58 |
| *Nylanderia* sp2 (Myrmicinae) | - | 0.21 | 0.21 |
| *Pheidole flavens* (Myrmicinae) | 0.33 | - | 0.33 |
| *Pheidole radoszkowskii* (Myrmicinae) | 0.16 | 1.01 | 1.13 |
| *Pheidole* sp1 (Myrmicinae) | - | 1.07 | 1.07 |
| *Pheidole* sp2 (Myrmicinae) | 2.01* | 3.05* | 4.05* |
| *Pheidole* sp3 (Myrmicinae) | 0.33 | - | 0.14 |
| *Pheidole* sp4 (Myrmicinae) | 0.08 | - | 0.14 |
| *Pheidole* sp5 (Myrmicinae) | 0.33 | - | 0.06 |
| *Pogonomyrmex naegelli* (Myrmicinae) | - | 10.04* | 10.04* |
| *Pseudomyrmex termitarius* (Pseudomyrmicinae) | - | 0.15 | 0.15 |
| *Solenopsis* sp1 (Myrmicinae) | 0.91* | 1.27 | 1.97 |
| *Solenopsis* sp2 (Myrmicinae) | 0.33 | - | 0.14 |
| *Trachymyrmex* gr *urichi* sp1 (Myrmicinae) | - | 0.21 | 0.21 |

Asterisks (*) denote ant species that were present in the central core of highly interacting species.
