# Supplementary material for: Unmasking the architecture of ant–diaspore networks in the Brazilian Savanna
Source: PLoS One. 2018 Aug 8;13(8):e0201117. doi: 10.1371/journal.pone.0201117 (PMC6082530; doi:10.1371/journal.pone.0201117)
Supplement: S5 Table — General linear models (GLMs) examining whether the mandible or diaspore size was related to species strength (ant or diaspore/plant species, respectively), considering fruit consumption and diaspore removal network, separately. (DOCX) [file pone.0201117.s005.docx]

**S5 Table.**

|  | F value | N | *p* value |
| --- | --- | --- | --- |
| **Fruit consumption network** |  |  |  |
| *mandible size*ant species strength* | 0.0012 | 15 | 0.9723 |
| *diaspore size* plant species strength* | 0.0048 | 6 | 0.9482 |
| **Diaspore removal network** |  |  |  |
| *mandible size*ant species strength* | 0.0125 | 17 | 0.9126 |
| *diaspore size* plant species strength* | 0.5705 | 27 | 0.4571 |
